# The effects of the soluble guanylate cyclase stimulator riociguat on memory performance in healthy volunteers with a biperiden-induced memory impairment

**DOI:** 10.1007/s00213-018-4938-0

**Published:** 2018-06-07

**Authors:** Laura G. J. M. Borghans, Anke Sambeth, Jos Prickaerts, Johannes G. Ramaekers, Arjan Blokland

**Affiliations:** 1 Department of Neuropsychology and Psychopharmacology, Faculty of Psychology and Neuroscience, Maastricht University, Universiteitssingel 40, 6229 ER Maastricht, The Netherlands; 2 Department of Psychiatry and Neuropsychology, School for Mental Health and Neuroscience (MHeNS), Faculty of Medicine, Health & Life Science, Maastricht University, Universiteitssingel 50, 6229 ER Maastricht, The Netherlands

**Keywords:** sGC stimulator, Biperiden, Episodic memory, Cognition, cGMP, Muscarinic receptors

## Abstract

**Rationale:**

After stimulation with nitric oxide, soluble guanylate cyclase (sGC) produces cyclic guanosine monophosphate (cGMP), which stimulates an important signalling pathway for long-term potentiation (LTP). By upregulating cGMP, LTP could be stimulated and thereby enhancing memory processes. The present study investigated the effects of the sGC stimulator riociguat on cognition in healthy volunteers. Participants were pre-treated with and without biperiden, which impairs memory performance, to investigate the memory-enhancing effects of riociguat.

**Methods:**

Twenty volunteers participated in a double-blind placebo-controlled six-way crossover design with a cognitive test battery including the verbal learning task (VLT), *n*-back task, spatial memory test, the attention network test, and a reaction time task. Treatments were placebo and riociguat 0.5 mg, placebo and riociguat 1.0 mg, biperiden 2.0 mg and placebo, biperiden 2.0 mg and riociguat 0.5 mg and biperiden 2.0 mg and riociguat 1.0 mg.

**Results:**

Blood pressure was found to be decreased and heart rate to be increased after administration of riociguat. Cognitive performance was not enhanced after administration of riociguat. Biperiden decreased episodic memory on the VLT, yet this deficit was not reversed by riociguat.

**Conclusion:**

This supports the notion that biperiden might be a valuable pharmacological model to induce episodic memory impairments as observed in AD/MCI.

## Introduction

It is predicted that the number of people diagnosed with dementia will rise to 48.1 million in 2020 and 90.3 million by 2040 (Prince et al. 2013). Unfortunately, no disease-modifying treatments have been developed yet. There are some symptomatic treatment options (e.g. cholinesterase inhibitors and NMDA antagonists), but these have limited effects and are associated with undesirable side-effects. Therefore, studies investigating the mechanisms of memory are valuable/needed in developing new treatments.

Nitric oxide (NO) is an atypical messenger involved in several functions within the central nervous system. NO regulates neurotransmitter release, blood flow, cell proliferation and also learning and memory formation (Susswein et al. 2004). NO is an important signalling molecule for the induction of LTP where it acts as a retrograde signalling molecule that binds to soluble guanylyl cyclase (sGC) on the presynapse. When NO binds to sGC, the synthesis of intracellular cGMP is triggered. Subsequently, the elevated cGMP levels activate PKG (cGMP-dependent protein kinase); both PKG (Arancio et al. 2001) and cGMP (Son et al. 1998) play a role in the induction of hippocampal LTP. LTP is a long-lasting increase in the efficiency of synaptic transmission in the hippocampus (Bliss and Lomo 1973) and it is thought that this is an important mechanism for learning and memory. In addition to this, several preclinical studies suggest that cGMP in the hippocampus is involved in the early stages of memory consolidation in the rat (Bernabeu et al. 1997; Bollen et al. 2014).

Riociguat is a sGC stimulator which has been approved under the name Adempas for the treatment of pulmonary hypertension (PH) (Ghofrani et al. 2010). It promotes vasodilation and inhibits smooth muscle proliferation (Grimminger et al. 2009). Patients with PH often present a NO deficiency, which leads to an understimulation of sGC. Riociguat acts as a sGC stimulator with a double mechanism of action; it stimulates sGC directly and next to this sensitises sGC to NO by stabilising the NO-sGC binding (Garnock-Jones 2014). This successfully improves the NO deficiency in these patients by restoring the NO-sGC-cGMP pathway, which leads to an increased generation of cGMP. Intracellular cGMP plays an important role not only in vascular processes but also in the induction of LTP, as explained above. Therefore, riociguat may have a positive effect on the induction and maintenance of LTP and thereby may improve learning and memory performance.

To investigate the cognition-enhancing effects of riociguat in healthy young volunteers, the same cholinergic deficit model was applied as in the animal study. Biperiden has been found to reliably induce a memory deficit in healthy volunteers (Borghans et al. 2017; Sambeth et al. 2015; Wezenberg et al. 2005). In contrast to the well-known scopolamine model (Ebert and Kirch 1998; Klinkenberg and Blokland 2010), biperiden is a selective muscarinic M1/M4 receptor antagonist. M1 receptors are particularly found in structures that are related to learning and memory. Moreover, M1 receptors have been shown to modulate LTP (Dennis et al. 2016), which is relevant for testing riociguat.

This study investigated the effects of varying single doses of riociguat on cognition in healthy young adults in a double-blind placebo-controlled design. Furthermore, we tested the effects of riociguat in a memory deficit model by administering biperiden. Different cognitive domains were tested: verbal episodic memory, spatial memory, working memory, attention and psychomotor performance. It was hypothesised that participants pre-treated with biperiden will show lower scores on episodic memory tasks and that riociguat would reverse the biperiden-induced episodic memory impairment.

## Method

### Participants

Healthy volunteers were recruited from Maastricht University through poster advertisements. After giving informed consent, participants underwent a medical screening, consisting of a medical questionnaire and physical examination.

Exclusion criteria were past or current psychiatric, neurological, cardiac, gastrointestinal, haematological, hepatic, pulmonary or renal illness, as well as pregnancy, lactation and excessive alcohol consumption (intake > 20 units/week). Participants were also required to have a body mass index of 18.5–30.0 kg/m^2^. Subjects using any medication other than oral contraceptives, having a first-degree relative with a current or past psychiatric disorder, or the presence of sensory or motor deficits that could influence performance, were also excluded. Finally, only non-smoking participants were included.

All subjects were financially rewarded for their participation. The medical ethics committee of Maastricht University and the Academic Hospital Maastricht (The Netherlands) approved the study.

In total, 20 participants took part in the study. The mean age was 22.3 years (S.D. = 2.4, range 20–27) and 12 were female. Two participants did not complete all test sessions, one after the first test day due to recurrent medical problems and one after the fifth test day due to nausea. These participants were excluded from the statistical analysis.

### Study design and treatments

This study was a double-blind, placebo-controlled, six-way crossover design. The treatments were administered in a balanced order over 6 test days using a Latin square, separated by a washout period of at least 5 days. The study had the following treatment arms: placebo and riociguat 0.5 mg, placebo and riociguat 1.0 mg, biperiden 2.0 mg and placebo, biperiden 2.0 mg and riociguat 0.5 mg, biperiden 2.0 mg and riociguat 1.0 mg, double placebo.

Biperiden (Laboratorio farmaceutico S.I.T., Mede, Italy) is a muscarinic M1 antagonist used for the treatment of Parkinson symptoms. Peak plasma concentrations are reached 1–2 h after a single-dose administration. Common side-effects on the central nervous system are drowsiness, vertigo, headache and dizziness. Peripheral side-effects consist of blurred vision, dry mouth, mydriasis, impaired sweating, abdominal discomfort and obstipation. In this study, a dose of 2 mg was used, a dose well within the range of the recommended doses for biperiden. Furthermore, research has found that an oral dose of 2 mg impaired cognitive performance in healthy adults (Borghans et al. 2017; McShane et al. 2006; Sambeth et al. 2015; Wezenberg et al. 2005).

Riociguat is a drug approved for the treatment of PH. Peak plasma concentrations are reached around 1–1.5 h after a single 1-mg dose and the terminal half-life is around 7 h in healthy subjects and 12 h in patients (Frey et al. 2008). The most common side-effects seen in association with the use of riociguat are headache, dizziness, dyspepsia, peripheral oedema, nausea, diarrhoea and vomiting. Based on our experience with roflumilast in animals (Vanmierlo et al. 2016) and humans (Heckman et al. 2018; Van Duinen et al. 2018), the formula of Reagan-Shaw et al. (2008) was used to calculate the appropriate dose. Therefore, we suggested that a dose of 0.5 and 1.0 mg was sufficient to improve memory performance.

### Procedure

After inclusion in the study, the participants first performed a training session. During this session, all cognitive tests were practiced to familiarise the participants with the study procedures and minimise procedural learning effects.

All test days started with the assessment of the general status, participants filled in the questionnaires and vital signs were measured. Next, they received biperiden or a placebo with a glass of tap water (T0). This was followed by a waiting period in which the participant ate a light breakfast or lunch with water or tea without caffeine. At T30, the subject received riociguat or a placebo with a glass of tap water. At T90, additional questionnaires were filled in, vital signs and a blood sample were taken and the cognitive testing started. At the end of test performance, again vital signs and a blood sample were taken. See Table 1 for an overview of time after dose for each task.

**Table 1:** Order of the tests and the relative time to drug administration (TAD) at the start of each test

TAD	Treatment
T-10	Questionnaires | vital signs
*T0*	*Biperiden 2.0 mg or placebo*
T5	Standardised meal
*T30*	*Riociguat 0.5 mg, 1.0 mg or placebo*
T90	Questionnaires | pupil | vital signs | blood sample
T100	VLT immediate recall
T110	SMT immediate
T120	*N*-back tasks
T130	Break | questionnaires
T140	VLT delayed recall and recognition
T150	SMT delayed recognition
T155	Break
T158	ANT
T175	Simple and choice reaction time task
T185	Pupil | vital signs | blood sample

Italic was used to highlight the administration of the treatments

### Cognitive tasks

In the current experiment, a range of cognitive tests, questionnaires and physiological measures were used. The main outcome measure was the scores on the verbal learning task, which measures storage, consolidation and retrieval of episodic memory using a word list. Three additional memory/attention paradigms were used to measure different aspects of memory and attention to obtain a broader view on the effects of riociguat on cognition. Treatment effects may be due to impairment in motor processes; therefore, a fourth test that examines motor and mental response speed (simple and choice reaction time task) was used to examine potential beneficial effects on psychomotor activity. Additionally, vital signs (heart rate and blood pressure) and pupil size were measured, for safety reasons and to confirm drug activity. Furthermore, questionnaires assessed the mood state of the participant (Profile of Mood States) and possible side-effects (complaints questionnaire).

#### Verbal learning task

An adapted version of the Rey auditory verbal learning test (Lezak 1995; Riedel et al. 1999) was used to assess short- and long-term memory function for verbal information. In this test, a list of 30 monosyllabic words in English was presented on a computer screen for 1 s with an interval of 1 s. The words were presented three times in the same sequence, immediately after presentation of the sequence a free recall phase followed (immediate recall). Approximately 30 min after the third trial, the participant was asked to freely recall as many words as possible (delayed recall). Subsequently, a recognition test was presented, consisting of all former, familiar words and 30 new but comparable words. The words were shown on a computer screen for max. 1500 ms and participants were asked to rate whether they were presented in the learning trials by a ‘yes/no’ response. A new trial started 3500 ms after presentation of the previous word.

Each session, a different word list was presented to the participants. The order of the lists was balanced across assessments. Outcome measures were the number of words correctly recalled in the three immediate recall trials and delayed recall phase. In the recognition test, median reaction times were measured in milliseconds as well as the number of correct recognised words.

#### *N*-back task

In this test, cognitive control demands are manipulated by increasing working memory load over the range *n* = 0 to *n* = 2. In each condition, a sequence of *n* + 64 digits between 1 and 9 were presented one at a time in the centre of the display. The duration of each digit was 1500 ms and a response was required for each digit. The interval between digits was 500 ms. In the *n* = 0 condition, participants were required to judge whether the current digit was equal to a pre-specified digit. In the other *n*-back conditions (i.e. *n* = 1 and *n* = 2), participants were required to judge whether the current digit was the same as *n* positions back in the sequence. Participants responded by pressing buttons labelled ‘yes’ and ‘no’ using the left and right index fingers.

The order of the tasks were counterbalanced, provided that the *n* = 2 condition was always followed by the *n* = 0 condition. These procedures are chosen to ensure that participants have the chance to relax between conditions of higher load. There are 64 trials in each condition, which are preceded by 16 practice trials. In half of the trials, the current digit matches the *n*-back digit, whereas in the other half it does not. Participants will be instructed to respond as fast and accurately as possible.

Reaction time and accuracy was analysed for each of the three conditions (0-back, 1-back, 2-back).

#### Spatial memory task

The spatial memory task assesses spatial memory and is based on the object relocation task by Postma and colleagues (e.g. Kessels et al. 1999). It consisted of one immediate and a delayed condition. In the immediate condition, a set of 10 pictures was presented one by one on different locations within a white square on a computer screen. All pictures were everyday, easy-to-name objects, presented in grayscale (± .3.5 × 5 cm). Each picture was presented for 2000 ms with an inter-stimulus interval of 1000 ms. This was followed by a ‘relocation’ part, which consisted of the presentation of a picture in the middle of the screen, followed by a ‘1’ and a ‘2’ being presented on two different locations. The participants’ task was to decide where the picture was originally presented, in location ‘1’ or location ‘2’. The ‘1’ and ‘2’ remained on the screen until the participant responded. After relocation, which was accomplished by a button press, the next picture was presented followed by the ‘1/2’ choice option. This continued until all 10 pictures had been relocated. Thereafter, the next set of 10 pictures was presented. A total of six sets of 10 pictures were displayed. Thirty minutes later, participants performed the delayed version. The original locations were not presented again. Subjects immediately started with the relocation part of the task.

Outcome variables were the number of correct relocations, as well as the RT of relocating. For the recognition phase of the test, the median RT was used as a measure of speed of retrieval from long-term memory.

#### Attention network test

The attention network test (ANT) evaluates three functions of attention within a single task (Fan et al. 2002). Each trial of this task started with the presentation of a fixation cross in the middle of the computer screen. Participants were instructed to keep their eyes fixed on this cross throughout the test. Cues in the form of an asterisk were presented for 100 ms, after which a target arrow appeared 400 ms later. The target remained on the screen until the participant responded by a key press with their index finger corresponding to the direction of this target (i.e. left or right) or if no response was given for 1700 ms. The inter-stimulus interval was 3500 ms. Furthermore, the task consisted of three target conditions and four cue conditions. Targets (neutral, congruent or incongruent) could appear above or below the fixation cross. Cue conditions (no cue, centre cue, double cue or spatial cue) indicated that the target was about to be presented and only spatial cues provided information about the location of the impending target. Spatial cues could appear above or below the fixation cross, indicating with 100% validity where the target would be presented.

Outcome variables included differences between RTs reflecting efficiency of alerting (RT no cue–RT double cue), orienting (RT centre cue–RT spatial cue) and executive network (RT incongruent–RT congruent).

#### Reaction time task

This task contained three parts, measuring simple reaction time (SRT) first, choice reaction time (CRT) second and incongruent choice reaction time (ICRT) last. For all parts, the participant was instructed to keep the index finger of their dominant hand pressed on the red button of a six-button response box, unless they needed to respond. In the first part, the participant had to react as quickly as possible by pressing the button lighting up in the centre of the response box. Second, one of five possible buttons could light up. Again, the participant had to press the lighted button as quickly as possible*.* In the incongruent choice task, the same instructions were given as in the second part of the task, except now the participant had to press the button right next to the one that was lighted up.

The dependent variables of this task consisted of median initiation times (time needed to release the red button) and median movement times (time needed to move from the red button to the target button) of correct choices.

### Physiological measures

Blood pressure and heart rate were measured at baseline, T90 and T185. An estimation of the pupil size was made by measuring the ratio between pupil and iris.

### Questionnaires

#### Profile of Mood States

The Profile of Mood States (POMS) is a self-evaluation scale for short, alternating states (McNair et al. 1971). In this adapted version of the POMS, 32 bipolar sets of adjectives comprising five bipolar mood factors (anger, depression, fatigue, tension and vigour) were presented to the participant. The participant had to indicate to what extent these items were appropriate to his mood on a 0- to 100-mm scale.

For each of the five mood factors, the mean score was calculated. This score was compared between the baseline (t-10) and the test (T90 and T140), to examine whether the treatment changed their state.

#### Complaints and side-effects

In order to monitor whether the participants experienced any complaints or side-effects, a list consisting of 33 complaints was presented. Participants had to indicate whether they experienced a certain complaint (e.g. nausea) at a 4-point scale. A score of 0 stands for ‘I do not experience this complaint at all’ and a score of 3 stands for ‘I am experiencing this complaint strongly’.

Scores were compared between the baseline (t-10) and the test (T90 and T140), to examine whether the treatment induced any complaints and/or side-effects.

### Data analysis

Data were analysed using a repeated-measures analysis of variance (ANOVA). The treatments biperiden (biperiden, 2.0 mg or placebo), riociguat (riociguat, 0.5 mg, 1.0 mg or placebo) and time (different time points) were used as separate within-subjects factors to assess the effect of treatment and interactions between biperiden and riociguat. Greenhouse-Geisser correction was applied where necessary; however, the reported degrees of freedom in the result section were not corrected. Additionally, the data were screened for outliers. Analyses were performed separately for accuracy and reaction times.

For the verbal learning task (VLT), the following additional within-subjects factors were used: trial (1–3) was added for the immediate recall and stimulus type (familiar vs. new) was used for the recognition test. For the *N*-back, the within-subjects factor type (0, 1 and 2) was additionally used for analysis. With regard to the spatial memory task (SMT), the additional with-subjects factor was delayed (immediate vs. delayed recognition). Different measures of the ANT were analysed, namely alerting, orienting, executive and total. For the reaction time task (RTT), the different subtasks were analysed as different within-subjects factors. To correct for multiple comparisons, Bonferroni correction was applied on post hoc tests.

## Results

### Missing data

Out of the 20 participants that started the study, one participant did not complete one session due to nausea; this participant was excluded from analyses of the delayed measures of the VLT and SMT and the ANT and RTT. Another participant did not complete one session due to technical problems. Furthermore, due to technical reasons unrelated to the experiment, data was missing for one session of the VRT of two participants. These participants were excluded from the analysis of the specific tasks.

### Behavioural results VLT and VRT

**Fig. 1:**
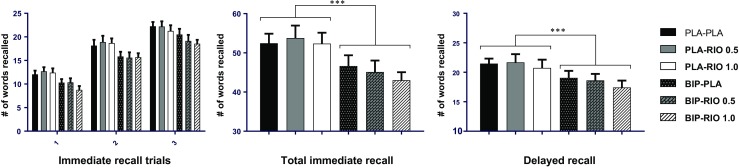
Mean number of words recalled during each immediate recall trial (left). The total number of words recalled during the three immediate recall trials (middle). The number of words recalled during the delayed recall after 30 min (right). Data represent mean and SEM. Biperiden significantly (*p* < 0.001) impaired performance in both the immediate and delayed recall task. PLA, placebo; RIO, riociguat; BIP, biperiden

Biperiden significantly impaired immediate recall, *F*(1,19) = 23.65, *p* < 0.001; participants significantly recalled fewer words after biperiden than after placebo (see Fig. 1). Riociguat, however, did not affect immediate recall, *F*(2,38) = 0.59, n.s.. Additionally, there was no interaction between the two treatments for immediate recall, *F*(2,38) = 0.66, n.s., see Fig. 1.

Biperiden significantly impaired delayed recall, *F*(1,18) = 16.14, *p =* 0.001. Riociguat did not affect delayed recall (*F*(2,38) = 1.83, n.s.). Additionally, there was no interaction between the two treatments for delayed recall, *F*(2,36) = 0.21, n.s..

In the recognition task, the median reaction time did not change after any of the treatments, nor was any significant interaction found between conditions. However, a significant interaction was found for biperiden and stimulus type, *F*(1,15) = 8.47, *p =* 0.011. Simple main effects analysis showed faster reaction times for biperiden (*M* = 595, SD = 12.7) compared to placebo (*M* = 614, SD = 15.7) for familiar words (*p =* 0.017). Furthermore, participants responded faster to familiar words (*M* = 614, SD = 15.7) than new words (*M* = 621, SE = 14.5) in the biperiden condition (*p =* 0.013), but in the placebo condition no differences between old and new were found (*p* = 0.544). The amount of correct detections for old and new words did not differ between treatment conditions.

### Behavioural results *N*-back

For both accuracy and reaction times, no differences were found between different treatments. Main effect of type (*F*(2,36) = 6.98, *p =* 0.01) was found for accuracy. Post hoc tests revealed that the accuracy was better for the 0-back (*M* = 0.93, SE = 0.011) compared to the 2-back (*M* = 0.893, SE = 0.013). For reaction times, significant effects were found for type as well, *F*(2,36) = 34.20, *p* < 0.001. Post hoc tests revealed that the accuracy significantly decreased with increasing task difficulty.

### Behavioural results SMT

**Fig. 2:**
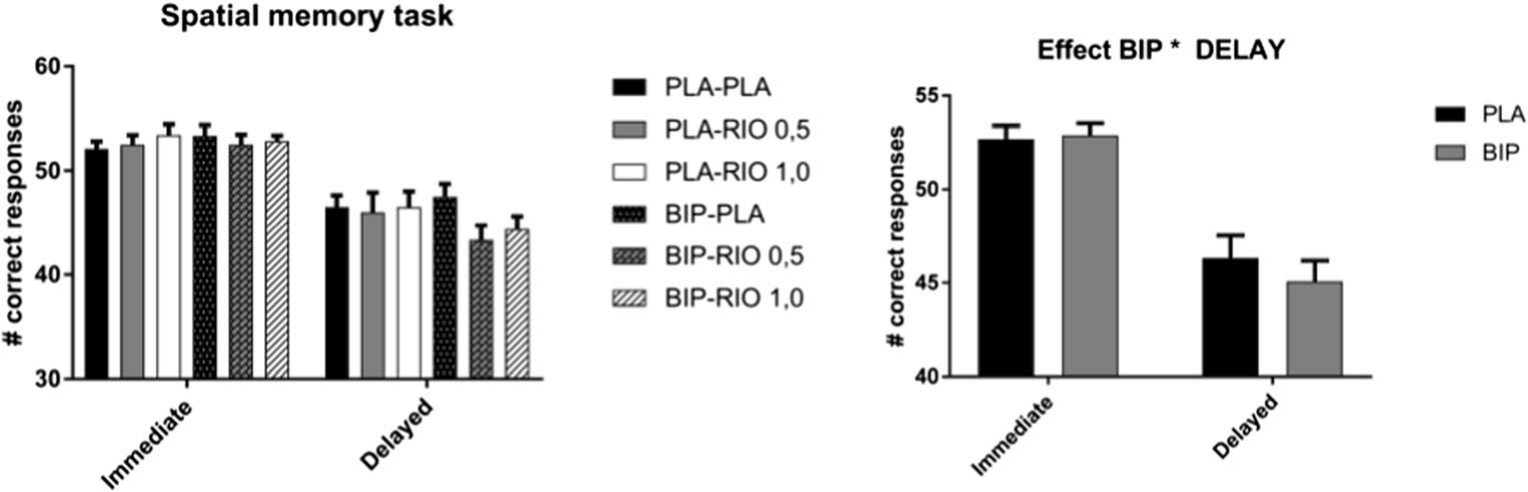
Mean correct responses (SEM) during the immediate and delayed trials of the SMT. Left: riociguat did not affect accuracy. A significant interaction between biperiden and delay was found. Right: No differences were observed between placebo and biperiden for immediate trials as well as for delayed trials. Participants had more correct responses for the immediate trials compared to the delayed trials for both the placebo and biperiden conditions. PLA, placebo; RIO, riociguat; BIP, biperiden

None of the treatments affected the accuracy during the SMT. A significant interaction between biperiden and delay was observed, *F*(1,17) = 4.59, *p =* 0.047 (see Fig. 2). Simple effects analysis revealed participants were more accurate on immediate trials compared to delayed trials in both placebo (*p* < 0.001) and biperiden (*p* < 0.001) condition. However, no significant differences between placebo and biperiden were observed for immediate (*p =* 0.76) and delayed trials (*p =* 0.206). This suggests a larger impairment of biperiden in the delayed trials compared to the immediate trials.

No treatment effects were found for reaction times. However, participants responded faster during the immediate trials compared to the delayed trials, *F*(1,17) = 5.95, *p* = 0.026.

### Behavioural results ANT

No significant results were found for biperiden on the alerting (*F*(1,17) = 1.06, n.s.), orienting (*F*(1,17) = 0.02, n.s.), executive (*F*(1,17) = 0.25, n.s.), or the total measure (*F*(1,17) = 0.05, n.s.). Also for riociguat, no significant differences could be observed (alerting: *F*(2,34) = 0.72, n.s.; orienting: *F*(2,34) = 0.199, n.s.; executive: *F*(2,34) = 0.78, n.s.; total: *F*(2,34) = 0.73, n.s.). The interaction between biperiden and riociguat was not significant (all *F*’s > 0.48).

### Behavioural results RTT

No significant results for the conditions were revealed after analysing the initiation times of the RTT (biperiden: *F*(1,17) = 1.77, *p =* 0.201; riociguat: *F*(2,34) = 0.98, *p =* 0.384; biperiden*riociguat: *F*(2,34) = 1.41, *p* = 0.257). A significant difference between the different parts of the task was observed, *F*(2,34) = 174.65, *p* < 0.001. Participants responded faster in the SRT compared to the CRT and ICRT and also faster in the CRT compared to the ICRT. Furthermore, the movement times did not differ significantly in the RTT for biperiden, riociguat or part (*F*’s < 2.11). The mean initiation and movement times based on median data are shown in Table 2 per condition.

**Table 2:** Mean scores (SEM) for the outcome variables of the attention network test (ANT), and reaction time task (RTT). For both tasks, no significant effects were found for condition, nor were there any interaction effects. *PLA*, placebo; *RIO*, riociguat; *BIP*, biperiden

BIP	PLA	PLA	PLA	2.0 mg	2.0 mg	2.0 mg
RIO	PLA	0.5 mg	1 mg	PLA	0.5 mg	1 mg
ANT effects (ms)						
Alerting	47 (4,4)	43 (5,6)	44 (5,2)	46 (4,6)	55 (4,7)	45 (4,7)
Orienting	37 (4,9)	42 (6,0)	27 (3,6)	36 (4,2)	36 (4,5)	37 (4,4)
Executive network	81 (4,7)	80 (8,0)	85 (6,6)	85 (6,2)	82 (5,2)	86 (6,0)
Total reaction time	432 (8,7)	437 (12,4)	437 (13,6)	435 (11,1)	432 (10,3)	442 (11,4)
RTT (ms)						
Initiation time						
SRT: IT	293 (8.3)	282 (5.6)	279 (7.2)	277 (6.5)	282 (5.7)	274 (6.6)
CRT: IT	322 (8.3)	318 (6.7)	311 (5.8)	314 (4.9)	316 (5.3)	313 (6.1)
ICRT: IT	367 (8.3)	366 (7.4)	365 (7.9)	362 (6.3)	369 (6.1)	364 (9.2)
Movement time (ms)						
SRT: MT	174 (8.6)	173 (10.7)	178 (8.8)	171 (9.8)	181 (10.8)	176 (9.9)
CRT: MT	187 (9.4)	178 (9.0)	180 (9.8)	178 (9.9)	185 (9.1)	182 (9.3)
ICRT: MT	189 (11.7)	186 (11.1)	182 (11.5)	184 (10.8)	186 (9.7)	188 (10.8)

### Physiological measures

**Fig. 3:**
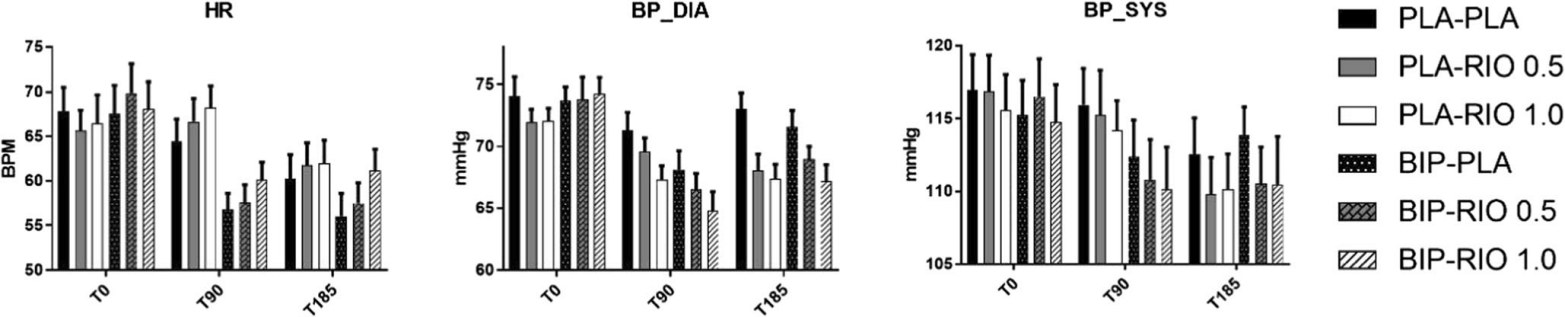
Physiological data mean blood pressure and heart rate measured at baseline, 90 min and 180 min after administration of biperiden (BIP) or placebo (PLA) and riociguat (RIO) or placebo. Heart rate decreased after BIP compared to PLA at T90 (*p* < 0.001) and T185 (*p =* 0.005). An increase of heart rate was found after RIO 1.0 mg at both T90 (*p =* 0.002) and T185 (*p =* 0.002). Blood pressure was significantly lower after BIP compared to placebo at T90 (*p <* 0.001). At T90, blood pressure significantly decreased after RIO 1.0 mg (*p =* 0.002) compared to PLA. Lastly, a significant decrease of blood pressure was found at T185 when RIO 0.5 mg (*p =* 0.011) and RIO 1.0 mg (*p <* 0.001) were compared with PLA

Figure 3 shows data on the different physiological measures.

Interaction effects were found for heart rate between biperiden and time (*F*(2,36) = 18.84, *p <* 0.001), as well as for riociguat and time (*F*(4,72) = 4.09, *p <* 0.05). Simple main effects analysis revealed that heart rate decreased after biperiden compared to placebo for T90 (*p* < 0.001) and the T185 (*p =* 0.005) measurement moment. Regarding riociguat, significant effects were found between placebo and riociguat 1.0 mg as heart rate increased on both T90 (*p =* 0.002) and T185 (*p =* 0.002).

For blood pressure, similar interaction effects were found between biperiden and time, *F*(2, 36) = 8.10, *p =* 0.001. Blood pressure was significantly lower after biperiden compared to placebo at T90 (*p <* 0.001). Additionally, a significant interaction was found between riociguat and time, *F*(4,72) = 3.03, *p <* 0.05. At T90, blood pressure significantly decreased after riociguat 1.0 mg (*p =* 0.002) compared to placebo. Lastly, a significant decrease of blood pressure was found at T185 when riociguat 0.5 mg (*p =* 0.011) and riociguat 1.0 mg (*p <* 0.001) were compared with placebo.

Pupil ratio increased with biperiden compared to placebo, *F*(1,18) = 8.26, *p =* 0.01. Riociguat did not affect pupil ratio.

### Questionnaire data

For the Profile of Mood States, significant interactions between biperiden and time were observed for depression (*F*(2,38) = 3.97, *p =* 0.032), anger (*F*(2,38) = 5.19, *p =* 0.015), fatigue (*F*(2,38) = 6.49, *p =* 0.006) and vigour (*F*(2,38) = 6.88, *p =* 0.010). Simple main effects showed that only for vigour a significant difference between placebo and biperiden was observed at T140 (*p* = 0.025). Other comparisons did not show significant effects between placebo and biperiden for the different mood scales on the different measurements. Riociguat did not affect any of the mood scales measured by the POMS.

Riociguat did not affect any of the adverse effects measured in Table 3. A significant interaction between biperiden and time (*F*(2,38) = 5.331, *p* = 0.010) for dry mouth showed that participants reported the feeling of dry mouth after biperiden compared to placebo at T90 (*p* = 0.005) and T140 (*p* = 0.022). Participants did not report any changes for other adverse effects, see Table 3.

**Table 3:** Mean scores (SEM) for the questionnaire data. Statistically, differences from placebo (*p <* 0.05) are denoted with an asterisk (*). *PLA*, placebo; *RIO*, riociguat; *BIP,* biperiden. Measurements were done at baseline, T90 and T140. At T140, a significant difference was found between placebo and biperiden. Furthermore, participants reported an increase in dry mouth after biperiden at T90 and T140

Biperiden	PLA	PLA	PLA	2.0 mg	2.0 mg	2.0 mg
Riociguat	PLA	0.5 mg	1 mg	PLA	0.5 mg	1 mg
Profile of Mood States						
Depression	75.23 (2.8)	77.21 (1.8)	78.81 (2.2)	77.68 (2.5)	76.33 (2.5)	77.88 (2.4)
	77.38 (2.8)	79.07 (1.8)	77.68 (2.8)	77.03 (2.5)	74.73 (2.9)	76.15 (2.8)
	78.61 (2.4)	78.43 (1.9)	76.57 (2.8)	77.36 (2.3)	76.41 (2.4)	76.16 (2.6)
Tension	76.08 (2.8)	76.78 (2.6)	79.14 (2.7)	76.96 (2.1)	77.62 (2.7)	75.34 (2.9)
	78.31 (2.6)	76.99 (2.6)	78.46 (2.6)	74.86 (3.2)	75.19 (3.3)	75.85 (2.8)
	77.03 (2.8)	75.26 (3.1)	75.87 (2.8)	74.58 (3.2)	75.05 (2.4)	74.76 (3.2)
Anger	77.81 (2.5)	80.63 (1.7)	81.16 (1.9)	81.59 (1.9)	79.33 (2.8)	80.50 (2.3)
	80.00 (2.2)	80.83 (1.9)	79.97 (2.4)	80.54 (1.9)	77.41 (2.6)	78.25 (2.6)
	80.65 (2.4)	80.11 (1.9)	78.68 (2.6)	79.89 (1.9)	78.94 (2.5)	78.31 (2.3)
Fatigue	69.46 (3.9)	70.39 (3.4)	72.25 (3.4)	72.58 (3.3)	72.02 (3.0)	72.86 (3.2)
	70.50 (3.6)	69.83 (2.9)	71.18 (4.1)	69.37 (3.3)	68.58 (3.6)	68.01 (3.9)
	72.97 (3.4)	73.58 (2.4)	69.23 (4.4)	70.54 (3.6)	70.47 (3.3)	68.90 (3.9)
Vigour	77.84 (3.3)	77.27 (3.5)	78.57 (3.2)	80.01 (2.8)	78.92 (3.0)	77.45 (3.2)
	76.12 (3.1)	77.08 (2.5)	76.63 (3.8)	75.87 (3.4)	72.68 (3.7)	73.97 (3.9)
	80.84 (2.8)	78.56 (2.7)	74.77 (4.1)	76.81 (3.5)*	73.67 (3.7)*	70.25 (4.0)*
Adverse effects						
Dry mouth	0*.*15 (0*.*11)	0*.*25 (0*.*10)	0*.*20 (0*.*12)	0*.*25 (0*.*12)	0*.*20 (0*.*12)	0*.*15 (0*.*08)
	0*.*15 (0*.*11)	0*.*10 (0*.*07)	0*.*05 (0*.*05)	0*.*35 (0*.*13)*	0*.*30 (0*.*13)*	0*.*40 (0*.*13)*
	0*.*25 (0*.*12)	0*.*15 (0*.*08)	0*.*10 (0*.*07)	0*.*45 (0*.*15)*	0*.*45 (0*.*14)*	0*.*40 (0*.*13)*
Sleepiness	0*.*45 (0*.*14)	0*.*30 (0*.*11)	0*.*60 (0*.*20)	0*.*25 (0*.*12)	0*.*35 (0*.*11)	0*.*35 (0*.*11)
	0*.*55 (0*.*17)	0*.*40 (0*.*11)	0*.*60 (0*.*21)	0*.*55 (0*.*15)	0*.*55 (0*.*15)	0*.*60 (0*.*15)
	0*.*35 (0*.*13)	0*.*30 (0*.*11)	0*.*70 (0*.*19)	0*.*45 (0*.*11)	0*.*50 (0*.*14)	0*.*55 (0*.*14)
Nausea	0*.*00 (0*.*00)	0*.*00 (0*.*00)	0*.*10 (0*.*07)	0*.*05 (0*.*05)	0*.*05 (0*.*05)	0*.*00 (0*.*00)
	0*.*00 (0*.*00)	0*.*00 (0*.*00)	0*.*05 (0*.*05)	0*.*05 (0*.*05)	0*.*10 (0*.*07)	0*.*00 (0*.*00)
	0*.*00 (0*.*00)	0*.*00 (0*.*00)	0*.*00 (0*.*00)	0*.*00 (0*.*00)	0*.*15 (0*.*11)	0*.*10 (0*.*07)
Headache	0*.*05 (0*.*05)	0*.*05 (0*.*05)	0*.*10 (0*.*07)	0*.*15 (0*.*08)	0*.*05 (0*.*05)	0*.*10 (0*.*07)
	0*.*15 (0*.*08)	0*.*05 (0*.*05)	0*.*15 (0*.*08)	0*.*15 (0*.*08)	0*.*10 (0*.*07)	0*.*05 (0*.*05)
	0*.*20 (0*.*09)	0*.*20 (0*.*09)	0*.*15 (0*.*08)	0*.*05 (0*.*05)	0*.*10 (0*.*07)	0*.*05 (0*.*05)
Dizziness	0*.*05 (0*.*05)	0*.*05 (0*.*05)	0*.*05 (0*.*05)	0*.*00 (0*.*00)	0*.*05 (0*.*05)	0*.*00 (0*.*00)
	0*.*05 (0*.*05)	0*.*05 (0*.*05)	0*.*05 (0*.*05)	0*.*20 (0*.*16)	0*.*15 (0*.*08)	0*.*05 (0*.*05)
	0*.*15 (0*.*08)	0*.*05 (0*.*05)	0*.*05 (0*.*05)	0*.*00 (0*.*00)	0*.*10 (0*.*07)	0*.*15 (0*.*08)
Fatigue	0*.*15 (0*.*08)	0*.*15 (0*.*08)	0*.*40 (0*.*15)	0*.*20 (0*.*12)	0*.*15 (0*.*08)	0*.*10 (0*.*07)
	0*.*25 (0*.*12)	0*.*15 (0*.*08)	0*.*30 (0*.*15)	0*.*15 (0*.*11)	0*.*25 (0*.*12)	0*.*35 (0*.*13)
	0*.*10 (0*.*07)	0*.*20 (0*.*09)	0*.*40 (0*.*18)	0*.*25 (0*.*12)	0*.*25 (0*.*16)	0*.*30 (0*.*13)
Drowsiness	0*.*00 (0*.*00)	0*.*05 (0*.*05)	0*.*05 (0*.*05)	0*.*00 (0*.*00)	0*.*05 (0*.*05)	0*.*00 (0*.*00)
	0*.*05 (0*.*05)	0*.*15 (0*.*08)	0*.*20 (0*.*09)	0*.*35 (0*.*17)	0*.*25 (0*.*12)	0*.*15 (0*.*08)
	0*.*05 (0*.*05)	0*.*05 (0*.*05)	0*.*20 (0*.*09)	0*.*15 (0*.*08)	0*.*05 (0*.*05)	0*.*20 (0*.*09)

## Discussion

In this study, the effects of riociguat on different cognitive tasks were examined in healthy young volunteers. Riociguat did not affect any of the cognitive tasks measuring episodic memory, working memory, spatial memory, attention or psychomotor performance. Blood pressure decreased and heart rate increased after administration of riociguat as could be expected from its vasodilatory properties. In addition, riociguat did not reverse the biperiden-induced memory impairment. The current experiment again showed that biperiden impairs memory on the visual verbal learning task, but did not affect working memory, spatial memory, attention or psychomotor performance.

Phosphodiesterase-inhibitors (PDE-I) having cGMP as a substrate have shown positive effects on memory performance in animals and in one human study (Prickaerts et al. 2004; Shim et al. 2014; van der Staay et al. 2008). Riociguat targets the same molecular signalling cascade, albeit via a different enzyme (Mittendorf et al. 2009). Since both sGC stimulation and PDE5-I lead to increased cGMP levels, it could be assumed that both enhance LTP and thereby memory performance. However, an alternative mechanism through which riociguat could exert its effect relates to its vasodilatory properties. Riociguat is a drug that has been developed to treat hypertension in patients with PH because of its vasodilatory effect.

However, no cognitive effects were found after riociguat. This lack of efficacy may be related with the low brain penetration of the blood-brain barrier by riociguat as described in the European Public Assessment Report only exhibits a low penetration (European Medicines Agency 2014). Although we based the doses on the effects in animal studies, it cannot be excluded that another dose could be effective. Finally, it could be that the cGC mechanism may not interact with the cholinergic M1 receptor and that therefore deficit could not be restored.

The findings regarding the biperiden-induced memory impairment found in this study are in line with the results from other studies (Borghans et al. 2017; Sambeth et al. 2015). In the study of Sambeth et al. (2015), biperiden impaired recall on the immediate and delayed VLT and impaired accuracy on the SMT. In the study of Borghans et al. (2017), only the delayed recall of the VLT was impaired after biperiden. Furthermore, this study again conforms that biperiden is specific to episodic memory, as the results only show significant effects on measures of episodic verbal memory. No effects of biperiden were observed on the *n*-back tasks, SMT and ANT. This profile possibly resembles the impairments that are seen in (amnestic) mild cognitive impairment (MCI), where episodic memory is impaired but other cognitive measures are not impaired or at least to a lesser extent (e.g. Döhnel et al. 2008; Petersen 2004). As patients that suffer from MCI have an increased risk to convert into AD later (Bruscoli and Lovestone 2004), the biperiden-induced memory deficit model may apply to that patient group as well. Taken together, this study further supports the notion that biperiden might be a valuable pharmacological model to induce episodic memory impairments as observed in AD/MCI.

In this study, the goal was to examine whether riociguat could improve memory in healthy participants and whether it could reverse a biperiden-induced impairment. Riociguat did not improve the memory performance in human subjects. Biperiden specifically impaired episodic verbal memory, but riociguat did not reverse this effect. It needs to be demonstrated whether the null effect of riociguat in humans is related to low brain penetration, and/or inappropriate dosing.
